# How does safety netting for lung cancer symptoms help patients to reconsult appropriately? A qualitative study

**DOI:** 10.1186/s12875-022-01791-y

**Published:** 2022-07-20

**Authors:** Georgia B. Black, Sandra van Os, Cristina Renzi, Fiona M. Walter, Willie Hamilton, Katriina L. Whitaker

**Affiliations:** 1 Institute of Epidemiology and Health Care, University College London, 1-19 Torrington Place, London, WC1E 6BT UK; 2 Wolfson Institute of Population Health, Barts and The London School of Medicine and Dentistry, Queen Mary University of London, London, EC1M 6BQ UK; 3 Department of Public Health and Primary Care, University of Cambridge, Cambridge, CB1 8RN UK; 4 University of Exeter Medical School, University of Exeter, Exeter, EX1 1SU UK; 5 School of Health Sciences, University of Surrey, Guildford, GU2 7YH UK

**Keywords:** Lung cancer, Early diagnosis, Primary care, Safety netting, Diagnostic uncertainty

## Abstract

**Background:**

Safety netting in primary care is considered an important intervention for managing diagnostic uncertainty. This is the first study to examine how patients understand and interpret safety netting advice around low-risk potential lung cancer symptoms, and how this affects reconsultation behaviours.

**Methods:**

Qualitative interview study in UK primary care. Pre-covid-19, five patients were interviewed in person within 2–3 weeks of a primary care consultation for potential lung cancer symptom(s), and again 2–5 months later. The general practitioner (GP) they last saw was interviewed face-to-face once. During the covid-19 pandemic, an additional 15 patients were interviewed only once via telephone, and their GPs were not interviewed or contacted in any way. Audio-recorded interviews were transcribed verbatim and analysed using inductive thematic analysis.

**Results:**

The findings from our thematic analysis suggest that patients prefer active safety netting, as part of thorough and logical diagnostic uncertainty management. Passive or ambiguous safety netting may be perceived as dismissive and cause delayed reconsultation. GP safety netting strategies are not always understood, potentially causing patient worry and dissatisfaction. Telephone consultations and the diagnostic overshadowing of COVID-19 on respiratory symptoms impacted GPs’ safety netting strategies and patients’ appetite for active follow up measures.

**Conclusions:**

Safety netting guidelines do not yet offer solutions that have been proven to promote symptom vigilance and timely reconsultation for low-risk lung cancer symptoms. This may have been affected by primary care practices during the COVID-19 pandemic. Patients prefer active or pre-planned safety netting coupled with thorough consultation techniques and a comprehensible diagnostic strategy, and may respond adversely to passive safety netting advice.

**Supplementary Information:**

The online version contains supplementary material available at 10.1186/s12875-022-01791-y.

## Background

Safety netting is considered an important intervention for managing low-risk symptoms of cancer [1, 2], particularly in the UK where it forms part of the government guideline for suspected cancer management [3]. Safety netting refers to actions taken and advice given to patients by healthcare practitioners about how to monitor and re-seek help for new, recurrent, persistent, or worsening symptoms, which may benefit patients in terms of disease stage at diagnosis, treatment options and survival [4]. Safety netting is a high-volume activity, with GPs reporting in a qualitative study that they use some form of safety netting at the end of almost every consultation [5]. Indeed, an audit of patient records showed that safety netting is recorded in 44% of all patient contacts where a cancer is eventually diagnosed [6]. Safety netting is particularly important in the diagnostic management of lung cancer, where low predictive value symptoms such as cough and tiredness are the most common first complaints that patients present to primary care with [7, 8]. Safety netting is transactional [5], and is not effective if the patient does not hear or understand the advice they are given by the primary care healthcare professional (HCP) or if the advice is insufficiently specific [9, 10]. Heyhoe et al. [11] suggest that, as part of effective safety netting, HCPs and patients should work together to develop and agree strategies that encourage sharing of symptom monitoring, re-appraisal and feedback, which will aid diagnosis of cancer at an early stage [12].

Poorly communicated safety netting advice may be worse than none; patients may delay reconsultation for lung symptoms by several months if they perceive that symptoms have been initially attributed to a benign cause, for example chronic obstructive pulmonary disease [13–16].

Best practice guidelines for safety netting have been developed through consensus [17] and evidence synthesis [10], although HCPs may deviate from this in practice [1, 18]. There is some evidence to suggest that patients prefer clear directives, for example setting a specific timescale for reconsultation rather than an open‐ended invitation [19]; however, there is no evidence about whether patients understand or attend to safety netting advice, nor how this affects reconsultation behaviours.

This study used a qualitative dyadic (paired) design to understand how patients responded to safety netting in primary care for low predictive value symptoms related to lung cancer. These symptoms included cough, anaemia, fatigue, shortness of breath, chest pain, weight loss or appetite loss. We aimed to capture the effect of safety netting on the way patients judge their symptoms and consider (re)seeking help in primary care over time.

## Method

This was a qualitative thematic analysis study. The analysis was based on interviews with patients who had recently had a GP consultation for a low predictive value symptom related to lung cancer. A small subsample of the interviews was dyadic, incorporating a separate interview with the patients’ GP and a follow up interview. The data were collected between February 2019 and June 2020.

### Recruitment

Individuals were eligible for the study if they were over 40 years old, and had seen their GP within the last 2–3 weeks for symptoms which included cough, anaemia, fatigue, shortness of breath, chest pain, weight loss or appetite loss. These were chosen in consultation with our GP research colleagues as examples of symptoms which would be likely to warrant safety netting from the GP rather than immediate referral for investigation. Exclusion criteria included a previous cancer diagnosis.

Sample size was derived in relation to norms for qualitative research [20]. We recruited 20 participants, purposively selected for low socioeconomic status assessed by postcode deprivation score. We purposively sampled for diversity in terms of ethnicity, gender and age. Three GPs and 20 patients (Table 1) were recruited purposively to achieve variation in age, gender, geographic spread and ethnic background. The first five patient participants were recruited in general practices in deprived areas (lowest 30% SES) by local National Institute for Health Research Clinical Research Network research nurses. Patients meeting inclusion criteria were given a study recruitment pack and encouraged to contact the researcher if interested in participating. Once patients were recruited, the GP they saw most recently was also approached for interview. Participants gave written informed consent before interview.

**Table 1:** Participant demographics and interview characteristics

**Patient characteristics**		
**Gender**	Female	13
	Male	7
**Age**	Range	40–69
	Mean	50.9
**Ethnicity**	White	13
	Black	6
	Asian	1
**Presenting problems (some participants reported more than 1)**	Fatigue	14
	Cough	11
	Shortness of breath	6
	Appetite loss	2
	Anaemia	2
	Chest pain	1
	Weight loss	1
	Difficulty sleeping	1
**Deprivation score by postcode**	Most deprived 5%	2
	Most deprived 10%	8
	Most deprived 20%	6
	Most deprived 30%	4
**GP characteristics**		
**Gender**	Female	2
	Male	1

Due to COVID-19, the recruitment strategy was amended in March 2020. A specialist recruitment agency, Taylor McKenzie, recruited 15 participants using a database of potential research participants and by approaching patient support groups. A screening questionnaire ensured participants had presented to their GP within the last 2–3 weeks with a low predictive value symptom related to lung cancer and were also from the lowest 30% SES. Verbal informed consent was audio recorded.

### Interview procedure

The first five participants were interviewed face-to-face twice in a private room at the practice, once within 2–3 weeks of the consultation and again 2–5 months later. This may have affected the participants’ recollections through recall bias. Patients were offered the choice of being interviewed at home, at their GP practice or at the university. Their GPs were interviewed subsequently face-to-face in their consultation room. The remaining 15 patient participants were interviewed once only via telephone. Their GPs were not interviewed or contacted in any way. Before starting the interview, the interviewer made sure that the participant had recently presented to their GP with a potential lung cancer symptom. Flexible interview topic guides (Additional file 1: Appendix 1) were developed using published literature and feedback from patient representatives. All interviews were conducted by an experienced, female, qualitative social science researcher. Interviews were digitally recorded and transcribed verbatim.

### Analysis

Transcripts were imported into NVivo 11, qualitative data analysis software, and coded by two authors using an inductive approach. Author 1 and 2 (initials removed to allow blind review) initially read all transcripts, and author 1 coded all transcripts with a particular focus on GP-patient dyads. To ensure the coding represented the data it was discussed with author 2. After analysis of the five dyads, the remaining dataset of 15 single patient transcripts was coded. Initial codes were then grouped into potential themes, and a thematic map was produced. The themes were then discussed and finalised by author 1 and 2, who agreed that the final themes were supported by the data. The final analysis and a draft of this manuscript were checked by all co-authors.

## Results

### Theme 1: Patients prefer active safety netting strategies

Patients preferred safety netting that included advice and actions that actively promoted re-consultation or involved pro-active follow-up. We consider that an active approach is one that is pre-determined at the point of the consultation, for example, making a follow up appointment. This is in contrast to a passive approach, where the next action is not planned and is open to later judgement or interpretation.

[I appreciated the] information in the initial conversation with the receptionist, and the prompt contact from the doctor. And the advice, and him wanting to know, in a few days how I was feeling, was anything getting any worse, any more symptoms. (Patient 20, female, 57, telephone interview).

Active safety netting as part of a thorough and logical approach to managing their lung cancer-relevant symptoms allowed patients to understand the diagnostic strategy, in turn making the safety netting advice easier to understand. For example, in the example presented in Table 2 both the patient and GP describe the ‘logical’ steps the GP took to reach the diagnosis over several visits. In his first interview the patient described how the GP had addressed each of his concerns through a series of logical steps, in his second interview he reflected on how this approach had been reassuring.

**Table 2:** Thoroughness in consultation (Patient 4 and GP 2)

Patient 4, male 47 years old	GP2, female
Most important thing was she was able to listen. She listened to me when I said my symptoms were and then **she talked me through**, saying, “I’ve listened to your chest at the front. Your chest is clear. There’s nothing to see in your throat. Your nose is not blocked. So, let’s get these tests done.” So, the first consultation, “Let’s do these tests and make sure everything’s alright, blah, blah, blah.” **Everything that I said she was able to check it** and say, “This is this, this is this, this is this.” Then okay, if it wasn’t showing up as a regular cold and she couldn’t identify a virus as yet, “let’s do these tests to make sure it’s nothing else.’’ She also spoke to somebody on the “phone, not within the practice, but I think it was at [hospital] and confirmed things. So, it felt like everything she was doing was logical and that was…yeah, I was reassured. Especially when the tests came back and there was nothing untoward, so that was a relief	I think at the beginning he came in; he’d had a cough; I think he’d had some recent travel and I thought it was a chest infection hence we went for the antibiotic route. I think he then saw one of my colleagues, had a similar story, treatment and then it was this persistent cough at which point I was thinking oh what’s going on. **Is this something else or is it just an allergic cough?** I think that’s why I gave the steroids. I’m relieved to know that I’m true because I couldn’t …he was otherwise okay. He hadn’t lost weight; he hadn’t lost his appetite. He was otherwise stable, and I don’t think he’s a smoker, so I couldn’t. He was quite an unclear one because I couldn’t. From a cancer side yes, you’d be thinking he’s got a persistent cough and I think I did request a chest X-ray as well. So **that was my cover** but […] I think he was worried, and he was suffering more than anything. I don’t know, but I think it was more that he was getting frustrated that his cough wasn’t settling. I’m not sure we had any magic cures for it and that’s why he was coming back

Several patients commented that their telephone consultation was with a GP they had not met before, who had not read their notes and was unaware of their history. They were dissatisfied with the level of thoroughness in the consultation, were not sure that all possible diagnoses had been considered, and felt ‘unsafe’ as a result.

If I get on the phone and talk to a GP, and they work through my symptomology, I discuss some of my background, what's been happening with me, get some historical data, and to then come to a conclusion in terms of a prognosis. Had that been done, in that kind of environment, I suppose, I would have thought “You know what, they've taken the time to research what's going on with, happen with me historically”. (Patient 14, male, 50, telephone interview)

Patients who had attended a telephone consultation during the COVID-19 pandemic, where the focus was perhaps mostly on acute symptoms and/or ruling out COVID-19, found it difficult to remember everything they wanted to discuss with the GP without a thorough discussion of wider issues.

GPs reported that they used active safety netting with certain patients, but not others, particularly when their “level of worry is a bit higher” (GP 3).

Where your level of worry is a bit higher than I'd probably tend to either book people to come back or book a phone call or something. So that I'm following it up. And I guess obviously, there's you the sort of specific symptoms but depending on different types of cancers, so sort of people with back pain, say, which is one we see quite often so we tend to say to them, I guess that's not specifically cancer so much but the kind of looking out for, if they suddenly have signs of, because of weight loss, things like that. (GP 3, female)

Patients engaged with diagnostic uncertainty and management as part of a thorough and attentive consultation, especially when active steps were taken to promote re-consultation.

### Theme 2: Patients interpret passive or ambiguous safety netting strategies as dismissive

Patients interpreted passive or ambiguous safety netting (such as verbal instructions to come/call back if symptoms do not resolve without specific timeframes or pre-planned appointments) as dismissive, and a sign that the GP was uninterested in their problem. This was a particularly salient experience for patients who had a telephone consultation. For example, Patient 13 had a telephone consultation during the pandemic, and inferred that the GP was not “that interested”, despite their assurances that she should get in contact if her symptoms persisted:

I think I had the cough and the fatigue for about a week. And so that's when I rang up the doctor, and the doctor weren't really that interested. Told me to ring back if I got any worse. He was more concerned about my chest, but my chest was fine. I think he was going on the lines of this virus rather than anything else…yeah. Get in contact if it gets worse, or if it is serious phone the NHS, that was it! I felt a bit daft when I've come off the phone […] I wasn't offered anything. Not at all. (Patient 13, female, 48, telephone interview)

Some patients experienced feelings of shame following passive safety netting, and felt dismissed particularly when access to a blood test was restricted. These patients did not receive specific advice on how to deal with COVID-related disruptions to additional tests they may need.

And so, there is nothing they suggest "Oh, call me back at such and such at a later time and we'll check it." Obviously, the problems is right now I can't go and get a blood test. So, I guess that's made it problematic. Or a follow-up appointment. […] Yeah, it did feel rushed. This is what you're getting, and see you later. (Patient 10, female, 40, telephone interview)

GPs justified passive safety netting in consideration/management of cancer risk, but also in terms of lowering patient anxiety:

A lot of what we do in general practice is actually reassure the worried well and a lot of discussions about cancer safety netting is actually just doing the exact opposite. (GP 1, male).

GP 3 believed that being too specific in safety netting, e.g. explicitly naming red flag symptoms, could raise anxiety among patients at very low risk of cancer.

Once you explain the list of things to look out for that people start getting them more often. […] I don't think it's enough of a negative not to do it. But certainly, there are a few patients where almost anything, if you'd asked them about it, they'd, manage to find an example of it. (GP3, female).

This suggests that GPs may engage in passive or ambiguous safety netting to lower patient anxiety, although this was not reflected in patient experiences.

### Theme 3: The GP’s diagnostic strategy is not always known to patients

GPs described diagnostic strategies predominantly in relation to managing cancer risk, including some strategies not communicated to patients, such as heuristics about a patient’s characteristics (e.g. age), and future plans in the event that symptoms persisted. When patients were unaware or had not understood the GPs diagnostic strategy, there was often a feeling of concern or lack of resolution. Table 3 presents an example of misalignment between patient and GP, where the patient was unaware that the GP was ruling out anaemia [to exclude cancer] and did not feel reassured by blood tests and an x-ray as a diagnostic management strategy. She was, however, reassured following an MRI scan, which she felt was the only way to properly rule out cancer. The GP did not mention the patient’s MRI scan during the interview.

**Table 3:** Diagnostic strategy did not reassure patient (Patient 1 and GP 2)

Patient 1 (female, 62 years old)	GP 2 (female)
But he said there is nothing seriously going on, it’s just your nerve endings are a bit, sort of, getting old, I suppose, and we all get aches and pains as we age. So, I’m happy with that, because at least I know they’ve had a good look inside me, **to out-rule if it was cancer or anything else, which I was worried about** *[Interviewer] so did you ever discuss with the GP that you were concerned about that it might be cancer?* I did, yes, speak to the GP about it and, when I said I do belong to [private health insurance firm], so she wrote a referral letter […] **I suggested, myself, please may I have an MRI scan**, because X-rays can only pick up so many things, where an MRI can up a lot more, and a lot more detailed. So, yeah, I’m very glad I had it done; very glad	I did, so when I requested the bloods, I requested a bone profile as well and full blood count **to make sure there was no anaemia**. So yes, I did. But I didn’t feel, based on her history, that was likely to be cancer. **I did the tests to make sure we weren’t missing it**, but because she was otherwise well it didn’t fit with myeloma or anything like that. I still did all the investigations […] Yes, I think the fact that she was running a business and she looks after the grandchildren and she’s standing a lot all made me think well this is most likely getting to be mechanical back pain rather than something more sinister. So, I guess from the history what I was gathering was pointing me toward benign

Similarly, Patient 2 was not aware of the diagnostic management strategy that GP 1 was using to resolve her cough (Table 4). She was concerned that the GP did not appear aware of her medical history of whooping cough and silent reflux, and was not aware of the GP’s strategy in using a ‘trial-of-treatment’ approach (silent reflux) as well as waiting for potential parapertussis symptoms to resolve. The GP, on the other hand, thought that he had clarified this, and that they were working to an 8-week timeframe for re-consultation. In her second interview the patient reflected on her experience and explained that she felt that the GP had most likely reached the correct diagnosis, but that the diagnostic approach and next steps had been unclear to her throughout.

**Table 4:** Misunderstanding diagnostic strategy (Patient 2 and GP 1)

Patient 2 (female, 58 years old)	GP 1 (male)
*How are you feeling at the moment about the diagnosis?* R: I’m a bit confused how they are linked. Whether it is two separate conditions or…Some of the symptoms are…are similar. Yeah *Do you feel that the consultation, at the end, was it clearly linked to an action? Did you understand what was going to happen next?* R: Not really, because there was no suggestions for what I could do in the next sort of three or four weeks which would take me up to the end of the three-month period other than increasing the medication for the reflux, but not really anything about the whooping cough *So, it wasn’t clear to you what you should do if anything changed?* R: If I needed to rest or if I needed to drink different amounts. If maybe he’d known a little bit more about the acid reflux and other symptoms. That must’ve been somewhere back on my records because I went through quite a lot of testing and they found that I had a hernia, and nothing was sinister then. So, it’s just at the back of my mind. As he said, I can’t be absolutely certain. I know they do normally say that	So, there are viruses called parapertussis viruses that are very like whooping cough viruses that are continually circulating in the community and they frequently, not infrequently, can cause persistent coughs with paroxysms of coughs where you cough, cough, cough and can´t stop yourself coughing and they last ages. They last sort of three months and so with her I think with an eight-week history that was paroxysm and to me it was probably that or it´s silent reflux, so I've given her treatment for silent reflux and I'll see her again. To be honest if it´s parapertussis it would have cleared up by the time I see her again anyway so if it´s cleared up I'll just stop the PPI. If it´s not cleared up I'm guessing I'm probably going to be looking to refer her, probably just a respiratory referral

These findings indicate that safety netting is dependent on patient understanding of the diagnostic process in addition to comprehension of specific signs, symptoms and timeframes that should trigger later actions.

## Discussion

### Summary

This is the first study to our knowledge to examine how patients understand and interpret safety netting advice around potential low-risk lung cancer symptoms. Our results suggest that patients strongly prefer active management and follow up as part of safety netting, in line with definitions offered in previous studies [21]. Participants saw this as a thorough and logical approach to managing diagnostic uncertainty. Conversely, passive safety netting may be perceived as dismissive and provide a disincentive to reconsult. In contrast, the GPs in our study worried that active strategies, particularly mentioning red flag symptoms, may cause unnecessary concern. Our analysis also suggests that GPs do not always make their safety netting strategy understood, potentially resulting in a misalignment where the GP thinks they have made an active safety netting plan while the patient feels worried or dissatisfied by what they perceive to be a passive safety netting approach. Telephone consultations and the diagnostic overshadowing of COVID-19 on respiratory symptoms were likely to affect GPs’ safety netting strategies and patients’ appetite for active follow up measures.

### Strengths and limitations

The strength of this study is the focus on patient interpretation of real safety netting experiences. It is a limitation that we were not able to obtain more dyadic interview pairings to understand GPs’ aims and perspectives. We interviewed patients from a wide range of geographic areas, giving us a varied picture of patients at risk of lung cancer. Recruitment of GP-patient dyads was limited by the pandemic. The 15 patient participants recruited during the pandemic described significantly different, COVID-related, experiences from patients interviewed pre-pandemic. As much of the focus was on COVID-19, it was challenging to disentangle which actions were ‘routine’ safety netting and which were pandemic-related. However, given the likely persistence of remote consultation in primary care, our findings will have relevance as new guidance and local practices emerge.

### Comparison with existing literature

Our study builds on previous suggestions from a study of hypothetical safety netting preferences, which reported that patients need active reassurance around reconsultation [11]. Furthermore, our study demonstrates that patients are more likely to feel a sense of subjective ‘safety’ when safety netting is part of a robust and logical consultation, and re-accessing care is assured. This is in line with previous studies reporting patient perceptions of under-support and over-reassurance following all-clear diagnoses [16], and studies showing that patients’ perceptions of safety are associated with holistic and individualized care, and challenged by system barriers to healthcare access [22].

Our study extends recent research looking at the mismatch between safety netting in guidance and practice [1], by collecting empirical data about real consultations rather than hypothetical or consensus-driven designs. Our findings mirror previous interview studies with GPs highlighting variability and uncertainty in safety netting approaches, and worry about managing cancer risk amidst busy workloads [5, 18].

### Implications for research and/or practice

Future safety netting research should measure patient understanding and reconsultation behaviour, developing strategies that improve these outcomes without raising unnecessary anxiety. Future studies should conceptualise safety netting as a complex intervention for patient safety and diagnostic management, with the aim to achieve alignment between patient and GP about the presenting problem’s significance and next steps. Taking a Health Literacy Universal Precautions approach [23, 24] will improve the likelihood that patients understand advice and create an aligned diagnostic strategy [25].

Safety netting is a high-volume intervention which is crucial to early diagnosis of cancer. However, a missed cancer diagnosis is a relatively rare event, and should not be a measure of safety netting quality[26]. Quality improvement work should focus on measuring processes that indicate misunderstood safety netting advice or patient disempowerment, such as delays to reconsultation, missed tests, and unfulfilled prescriptions.

GPs report having a relatively low threshold for referring patients with lung cancer symptoms [27]; safety netting practices may vary for symptoms relating to other cancers, with differential experiences for patients.

## Conclusion

Diagnostic management of patients with low-risk lung cancer symptoms in primary care is a crucial mediator in promoting early diagnosis. Safety netting guidelines do not yet offer solutions that have been proven to promote symptom vigilance and timely reconsultation. Patients prefer active safety netting coupled with thorough consultation techniques and a comprehensible diagnostic strategy, and may respond adversely to passive safety netting advice.

## Supplementary Information

**Additional file 1:** **Appendix 1.** Interview discussion guides.

## Data Availability

The data sets generated and analysed during this study are available from the corresponding author on reasonable request. The approach taken for the study is detailed in the main text and could be reproduced in any similar qualitative interview study.
