# Correction: How does safety netting for lung cancer symptoms help patients to reconsult appropriately? A qualitative study

**DOI:** 10.1186/s12875-022-01808-6

**Published:** 2022-08-04

**Authors:** Georgia B. Black, Sandra van Os, Cristina Renzi, Fiona M. Walter, Willie Hamilton, Katriina L. Whitaker

**Affiliations:** 1 Institute of Epidemiology and Health Care, University College London, 1-19 Torrington Place, London, WC1E 6BT UK; 2 Wolfson Institute of Population Health, Barts and The London School of Medicine and Dentistry, Queen Mary University of London, London, EC1M 6BQ UK; 3 Department of Public Health and Primary Care, University of Cambridge, Cambridge, CB1 8RN UK; 4 University of Exeter Medical School, University of Exeter, Exeter, EX1 1SU UK; 5 School of Health Sciences, University of Surrey, Guildford, GU2 7YH UK

**Correction: BMC Primary Care 23, 179 (2022)**

10.1186/s12875-022-01791-y

Following publication of the original article [1], the authors identified the following errors in the published version.

•Fiona Walter should have a middle initial ‘M’ i.e. “Fiona M. Walter”

•Affiliation 2 should be “Wolfson Institute of Population Health, Barts and The London School of Medicine and Dentistry, Queen Mary University of London, EC1M 6BQ, UK”

The mistakes were rectified in this article and in the original article.

## References

[1] Black GB, van Os S, Renzi C (2022). How does safety netting for lung cancer symptoms help patients to reconsult appropriately? A qualitative study. BMC Prim Care.

