# Supplementary material for: How does safety netting for lung cancer symptoms help patients to reconsult appropriately? A qualitative study
Source: BMC Prim Care. 2022 Jul 20;23:179. doi: 10.1186/s12875-022-01791-y (PMC9298706; doi:10.1186/s12875-022-01791-y)
Supplement: Supplementary file 1 — Additional file 1: Appendix 1. Interview discussion guides. [file 12875_2022_1791_MOESM1_ESM.docx]

SUPPLEMENTARY MATERIAL

**Appendix 1: Safety netting and re-consultation: GP and patient perspectives**

Interview discussion guide – Patient interview 1

| Consultation | - Can you tell me about what happened when you went to see your GP? - What prompted you to go and see them? |
| --- | --- |
| GP advice | - What did the GP say? - How did you feel afterwards? - How did the GP respond after you had described your symptoms? - What did they say about what these symptoms may mean? - Tell me about anything else they said. - How did you feel about this? |
| Follow-up/re-appraisal | - How did you make sense of what was wrong? - What did you plan to do about it? - Have you seen with the GP since the consultation?   - If yes, what made you go back? - What were the most important things the GP said and did relating to your symptoms? - Is there anything that I haven’t asked you about that may be important? |
| Current health | - What’s happening with your health now? - How have you been managing it? - Have you had any other contact with the GP? - Have you seen any other medical professional/A&E since your consultation?   - If yes, what prompted this? |

Interview discussion guide – Patient interview 2

| Follow-up /re-appraisal | - Remembering our previous interview, you described X… How is that now? - How is your health otherwise?   - Treatment   - Help-seeking and information seeking   - Self-help - How do you make sense of that consultation now? - What do you think was wrong with your health? - Have you seen/had contact with the GP since the consultation?   - If yes, what made you go back? - And what about other medical professional/A&E?   If yes, what prompted this? |
| --- | --- |
| Current health | - What’s happening with your health now? - How have you been managing it? - How are you planning to move forward? - What were the most important things the GP said and did relating to your symptoms? - Is there anything that I haven’t asked you about that may be important? |

Discussion guide – GP interview

| Safety netting generally | - How does safety netting generally work in your practice?   - And safety netting for cancer symptoms in particular? - What are the objectives of safety netting? - Tell me about a typical situation where safety netting works well   - And not so well - How do you record or document the safety netting you do? - Is there anything else about safety netting that is important for me to know? |
| --- | --- |
| Safety netting paired patient | - Thinking back to patient X, what happened during the consultation? - Tell me about the safety netting - Objectives, able to deliver required safety netting, why? - Was there anything that made this consultation/patient different? - What happened next? - Is there anything else about this patient/consultation that you would like to share? |
